# Hantavirus infection in human and rodents in central highlands and southern Vietnam during 2006-2009

**Published:** 2011-01-10

**Authors:** Vu Thi Que Huong, Vu Dinh Luan, Kumiko Yoshimatsu, Midori Taruishi, Le Nhi, Endo Rika, Vo Thi Huong, Cao Minh Thang, Hoang Kim Loan, Jiro Arikawa

**Affiliations:** 1 Department of Microbiology and Immunology, Pasteur Institute of Ho Chi Minh City, Vietnam; 2 Department of Virology, Tay Nguyen Institute of Hygiene and Epidemiology, Dak Lak Province, Vietnam; 3 Institute for Animal Experimentation, Graduate School of Medicine, Hokkaido University, Japan

## 

From 2006 to 2009, a surveillance of the situation of hantavirus infection in Southern part and Highlands of Vietnam was carried out based on the tested results of serum samples from 1,066 rodents representing 6 species and 245 *Suncus murinus*.

The results of tested rodent sera samples by ELISA, IFA and confirmed by Western Blot showed that the prevalence antibody to hantavirus was 16.76% from *R.norvegicus*, and 13.1% from *S.murinus*. The serotyping result by FRNT revealed that hantaviruses which circulate in Southern Vietnam belong to Seoul and TPMV serotype. RNA of hantavirus was detected from 3 lung tissues of *Rattus norvegicus* samples which are coded as CSG5, CSG11 and 24D12, all collected in Ho Chi Minh City (Sai Gon Habor and District 12). The sequencing and phylogenetic analysis on detected genes (partial small and medium segments) demonstrated the close genetic relationship with SEOV representatives found in Japan, Indonesia Singapore and Northern Vietnam.

The serological analysis revealed the circulation of hantavirus including Seoul virus (SEOV) from *Rattus.sp*, and Thottopalayam virus from *S. murinus* in Southern and Highlands of Vietnam. Besides, the detection of specific IgM and the neutralizing antibody against SEOV in patient indicated the first evidence of the circulation and transmission of SEOV from rodent to human.

